# Radiomics-Based Machine Learning in the Diagnosis of Type-B Aortic Dissection on Computed Tomography Images

**DOI:** 10.12669/pjms.41.11.12896

**Published:** 2025-11

**Authors:** Yifeng Shen, Xinyi Shi, Jianqi Ni, Qin Jin, Guoliang Wang, Jiajun Zou

**Affiliations:** 1 Yifeng Shen Department of Vascular surgery, The First Hospital of Jiaxing, Affiliated Hospital of Jiaxing University, Jiaxing, Zhejiang Province 314000, P.R. China; 2 Xinyi Shi Department of Radiology, The Second Hospital of Jiaxing, Jiaxing, Zhejiang Province 314000, P.R. China; 3 Jianqi Ni Department of Vascular surgery, The First Hospital of Jiaxing, Affiliated Hospital of Jiaxing University, Jiaxing, Zhejiang Province 314000, P.R. China; 4 Qin Jin Department of Vascular surgery, The First Hospital of Jiaxing, Affiliated Hospital of Jiaxing University, Jiaxing, Zhejiang Province 314000, P.R. China; 5 Guoliang Wang Department of Vascular surgery, The First Hospital of Jiaxing, Affiliated Hospital of Jiaxing University, Jiaxing, Zhejiang Province 314000, P.R. China; 6 Jiajun Zou Department of Radiology, The First Hospital of Jiaxing, Affiliated Hospital of Jiaxing University, Jiaxing, Zhejiang Province 314000, P.R. China

**Keywords:** Type-B aortic dissection, Computed tomography, Radiomics, Machine learning model

## Abstract

**Objective:**

To evaluate the value of a radiomics-based machine learning model in detecting Type-B aortic dissection (TBAD) on computed tomography (CT) images.

**Methodology:**

This retrospective analysis included one hundred records of patients with clinically diagnosed TBAD and one hundred records of non-TBAD patients treated at the First Hospital of Jiaxing from January 2010 to January 2024. Radiomics features were extracted from CT non-contrast images, and the least absolute shrinkage and selection operator (LASSO) was used to construct dimensionality reduction and prediction models. The diagnostic performance of the model was evaluated through receiver operating characteristic (ROC) curves.

**Results:**

Fifteen radiomics features were extracted from the training cohort. All eight machine learning-established radiomics models in the training cohort demonstrated good prediction accuracy, with area under the ROC curve (AUC) values exceeding 0.9 in the validation set. Among the three models compared, the AUC values of the nomogram were the highest in both the training and validation cohorts (0.991 [95% confidence interval (CI): 0.982-1.000] and 0.998 [95% CI: 0.993-1.000], respectively). The calibration curves of the nomogram in both cohorts were more closely aligned with the dashed line. The nomogram showed the highest clinical benefits in both training and validation cohorts.

**Conclusions:**

The predictive model established based on radiomics analysis of CT images demonstrates good predictive ability in recognizing TBAD.

## INTRODUCTION

Aortic dissection is an acute and severe cardiovascular disease, with an annual incidence of about 2.8/100000 in China alone.^1^,^2^ With the increase in the incidence rate of hypertension and atherosclerosis, the incidence of dissection has also increased significantly in recent years.^2^ Aortic dissection may lead to rupture of the aortic intima, allowing blood to flow into the aortic media and resulting in separation of the inner and outer membranes, as well as tearing along the longitudinal axis of the aorta, ultimately leading to the separation of true and false cavities.^3^ Aortic dissection often manifests as a sudden, intense pain and is associated with various life-threatening symptoms.^3^,^4^ Without timely (within 48 hours) treatment, aortic dissection has a mortality rate of up to 50%.^1^-^4^ Therefore, early diagnosis and treatment of aortic dissection can significantly reduce patient complications and mortality.

Based on the location, aortic dissection may be classified as Type-A, involving the ascending aorta, and Type-B, involving the descending aorta.^5^ Previous studies have shown that less than half of Type-B aortic dissection (TBAD) patients exhibit typical clinical symptoms, and the clinical misdiagnosis rate in such patients is high, often leading to serious complications and even death.^3^,^4^,^6^ However, there is currently a lack of accurate and efficient methods for the early diagnosis of TBAD. The existing diagnostic methods, such as computed tomography angiography (CTA) and digital subtraction angiography (DSA), have significant limitations. Although CTA is considered a first-line examination method for TBAD, patient compliance is not high due to factors such as popularity, cost, and potential increased risk of kidney disease.^7^ Similarly, while DSA remains the gold standard for diagnosing aortic dissection, it is not conducive to early disease diagnosis and is more suitable for intraoperative use due to its invasiveness and time lag.^8^

Radiomics, which combines artificial intelligence and big data imaging, offers advantages such as safety, universality, speed, and cost-effectiveness.^9^ Currently, developing imaging analysis technology to support clinicians in early and rapid disease detection has become a focus of research.^10^ This study aimed to use radiomics to construct a predictive model for the early diagnosis of TBAD. Such a model is crucial for developing individualized treatment plans and improving the overall prognosis of TBAD patients through early intervention.

## METHODOLOGY

Clinical records of one hundred patients clinically diagnosed with TBAD and one hundred non-TBAD patients, treated at Jiaxing First Hospital from January 2010 to January 2024, were retrospectively analyzed. All patients underwent computer tomography (CT) plain scan and CTA before surgery or thoracic aortic endovascular repair (TEVAR) treatment. The non-TBAD group included patients who underwent contrast-enhanced chest CT examinations at the hospital during the same period, with no signs of aortic dissection or aneurysm on imaging, no history of aortic or vascular intervention, and no significant cardiovascular comorbidities. All CT scans were acquired using the same 320-slice CT scanner and identical scanning protocol to ensure imaging consistency across groups.

### Ethical Approval:

The study was approved by the Ethics Board of the Jiaxing First Hospital (Approval number 2025-LY-030; Dated: July 11, 2025) and was conducted according to the Declaration of Helsinki principles for medical research involving human subjects. Due to the retrospective nature of the study, informed consent was waived.

### Inclusion criteria:

- Complete emergency CT plain scan and complete data within two weeks of onset.
- No serious accidents occurred during the acute phase.
- Has not undergone aortic surgery or TEVAR before the CTA re-examination of the aorta.
- HCT shows no history of major vascular intervention or surgical intervention before surgery.

### Exclusion criteria:

- Age < 18 years old or > 90 years old.
- Chronic phase of interlayer.
- Poor image quality and large analysis errors.
- Pregnant women.

### Selection and assignment of clinical features:

To construct a predictive model for the diagnosis and prognosis of TBAD, and to make the model more comprehensive and efficient, gender, age, weight, hypertension history, C-reactive protein (CRP), diabetes history, smoking history, D-dimer, fibrinogen, low-density lipoprotein, cholesterol, and other clinical traits were used. Considering the large amount of data and complex parameters required for this study, to improve computational efficiency and visual display, all parameters involved in the research were assigned specific values. If there was no hypertension, a value of “0” was assigned during classification; otherwise, a value of “1” was assigned.

### CT image scanning:

All patients underwent a Canon 320-row CT examination, with CT scans ranging from above the aortic arch to the bifurcation level of the lower segment of the abdominal aorta, including both plain and enhanced scans. The images were uploaded to the Picture Archiving and Communication System (PACS) in DICOM format, and all patient plain scan image data were downloaded from the PACS system.

### Outline of Region of Interest (ROI):

All CT plain scan images were retrieved from the PACS system of a single institution and acquired using a standardized protocol on Canon 320-row scanners. To ensure consistency across the dataset, the following preprocessing pipeline was applied before radiomics analysis: (1) B-spline interpolation-based resampling was performed to convert all images to isotropic voxel spacing of 1×1×1 mm^3^; (2) grayscale normalization was applied to standardize Hounsfield Unit distributions and mitigate scanner-induced intensity variations; (3) no additional denoising filters were applied, but artifacts were minimized by careful manual delineation of ROIs by trained radiologists, excluding motion artifacts and irrelevant structures. This approach ensured robust feature extraction and minimized inter-image variability. A radiologist with three years of CT diagnostic experience, blinded to the disease, drew a region of interest (ROI) along each layer of the aorta and fused the layers to generate a 3D contour. Finally, another physician with three years of experience in vascular surgery conducted a review.

### Radiomics feature extraction:

To eliminate the influence of image differences, resampling was first performed. Radiomics features were extracted from images using Pyradiomics in Python. The feature parameters included: first-order features, shape features (shape 2D/3D), gray-level run length matrix (GLRLM), gray-level dependence matrix (GLDM), gray-level size zone matrix (GLSZM), neighborhood gray tone difference matrix (NGTDM), gray-level co-occurrence matrix (GLCM), and filtering features.

### Screening of radiomics features:

Initially, invalid or non-informative features were excluded. Missing values were imputed using mean substitution, and all features were standardized via Z-score normalization. Feature selection and model construction were conducted solely on the training cohort, while the independent test cohort was used exclusively for internal validation. To mitigate the risk of overfitting associated with high-dimensional radiomics data, a nested 10-fold cross-validation framework was employed. Within each outer fold, feature selection was performed on the training subset using the least absolute shrinkage and selection operator (LASSO) regression, coupled with inner 10-fold cross-validation for optimal λ selection. Feature selection was nested within each fold to prevent information leakage. Only features with non-zero coefficients were retained for model training, and an average of 12-15 stable features were selected across folds. Additionally, L1 regularization was applied during model training to enhance generalizability and interpretability by choosing the most discriminative predictors. To control potential confounding from imbalanced clinical variables, stratified random sampling was used to divide the dataset into training and validation cohorts in a 7:3 ratio. The distribution of key clinical variables (e.g., age, sex, blood pressure, diabetes history, D-dimer) was statistically comparable across subsets, confirming the baseline homogeneity.

### Construction and validation of predictive models:

This study incorporated the training dataset into eight machine learning models, including logistic regression, decision trees, support vector machines (SVMs), and adaptive boosting (AdaBoost). To construct the joint model (nomogram), a feature-level fusion approach was adopted. Specifically, radiomics features selected via LASSO regression were concatenated with clinically relevant features-such as D-dimer levels and systolic blood pressure-to form a combined feature vector. This vector was input into a multivariate logistic regression model, which internally assigned optimized weights to each feature through maximum likelihood estimation. Finally, receiver operating characteristic (ROC) curves were generated to evaluate the diagnostic performance of each model. The area under the ROC curve (AUC) and its 95% confidence interval (CI), as well as sensitivity, specificity, positive predictive value, negative predictive value, accuracy, and F1 score, were calculated for both the training and validation sets. A nomogram model was built to visualize abstract regression models and evaluate them using calibration and decision curves.

### Statistical analysis:

Statistical analysis was conducted using IBM SPSS Statistics 23.0 software. Count data were analyzed using the chi-square test or Fisher’s exact test. Measurement data that conformed to normal distribution were represented by mean ± standard deviation (SD) and Student’s t-test. Data that did not conform to normal distribution were expressed by median (upper quartile, lower quartile), and the Mann-Whitney U test was used. The difference is statistically significant when *P* < 0.05.

## RESULTS

As shown in Table-I, there were significant differences in the age, D-dimer levels, calcification ingression, internal displacement of intimal film, pleural and abdominal effusion blood, and calcification of the aortic arch between TBAD and non-TBAD patients (P<0.05).

All other parameters, such as the levels of fibrinogen, low-density lipoprotein, cholesterol, c-reactive protein (CRP), high attenuation area, pericardial effusion blood, calcification of ascending aorta, calcification of descending aorta, sex, as well as the history of hypertension, diabetes, and smoking were comparable in the two groups (P>0.05) (Table-I).

**Table-I:** Comparison of clinical data between TBAD group and non TBAD group.

Index	Non-TBAD group (n=100)	TBAD group (n=100)	P
Age (years), mean±SD	67.23±12.02	56.31±14.17	<0.001
D-dimer (ng/ml), mean±SD	1996.96±3533.95	4585.25±5031.07	<0.001
Fibrinogen (g/L), mean±SD	3.48±0.98	3.63±1.73	0.522
Low density lipoprotein (mmol/L), mean±SD	2.71±0.84	2.62±0.68	0.487
Cholesterol (mg/dL), mean±SD	4.32±0.97	4.22±0.83	0.505
CRP (mg/L), mean±SD	17.44±31.95	28.48±50.23	0.124
Calcification ingression (yes), n(%)	14(20.29)	36(50.70)	<0.001
Internal displacement of intimal film (yes), n(%)	17(24.64)	54(76.06)	<0.001
High attenuation area (yes), n(%)	11(15.94)	18(25.35)	0.244
Pericardial effusion blood (yes), n(%)	2(2.90)	5(7.04)	0.461
Pleural and abdominal effusion blood, (%)	1(1.45)	16(22.54)	<0.001
Calcification of ascending aorta (yes), n(%)	16(23.19)	7(9.86)	0.057
Calcification of aortic arch (yes), n(%)	47(68.12)	33(46.48)	0.016
Calcification of descending aorta (yes), n(%)	56(81.16)	49(69.01)	0.143
Male (yes), n(%)	56(81.16)	58(81.69)	0.891
hypertension (yes), n(%)	40(57.97)	48(67.61)	0.315
diabetes (yes), n(%)	8(11.59)	5(7.04)	0.524
smoke (yes), n(%)	16(23.19)	16(22.54)	0.913

CRP: C-reactive protein

**Fig.1:**
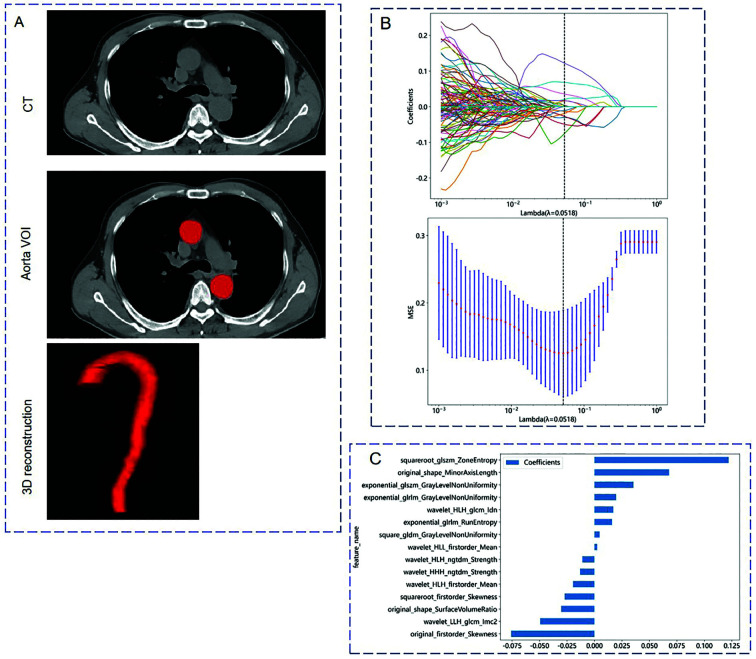
Workflow of Radiomics Analysis. (A) Two radiologists segment the CT images to manually depict the volume of interest (VOI) of the aorta at the voxel level. Then, radiomics features were extracted from the aortic mask using Pyradiomics. (B) The curves of different colors in the figure represent the trajectory of the variation of each independent variable coefficient. The vertical axis represents the coefficient values, and the upper horizontal axis represents the number of non-zero coefficients in the model at this time. (C) The LASSO regression algorithm selected 15 radiomics features based on the training queue.

A total of 1561 radiomics features were extracted on the PyRadiomics platform. The Lasso regression was used to reduce the dimensionality of radiomics features from 140 samples in the training cohort. A 10-fold cross-validation was employed to determine the optimal lambda value of 0.0518, resulting in 15 non-zero coefficient feature parameters (Fig.1).

**Fig.2:**
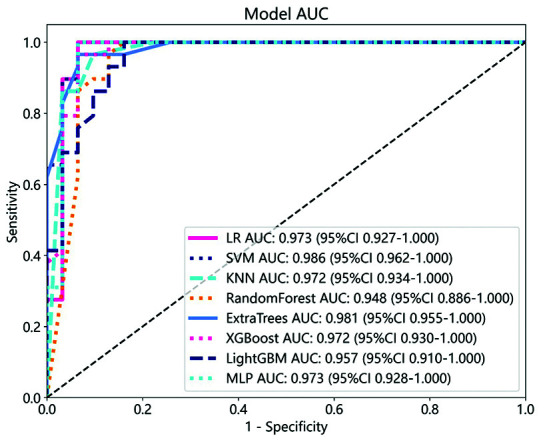
ROC curve of validation cohort.

**Fig.3:**
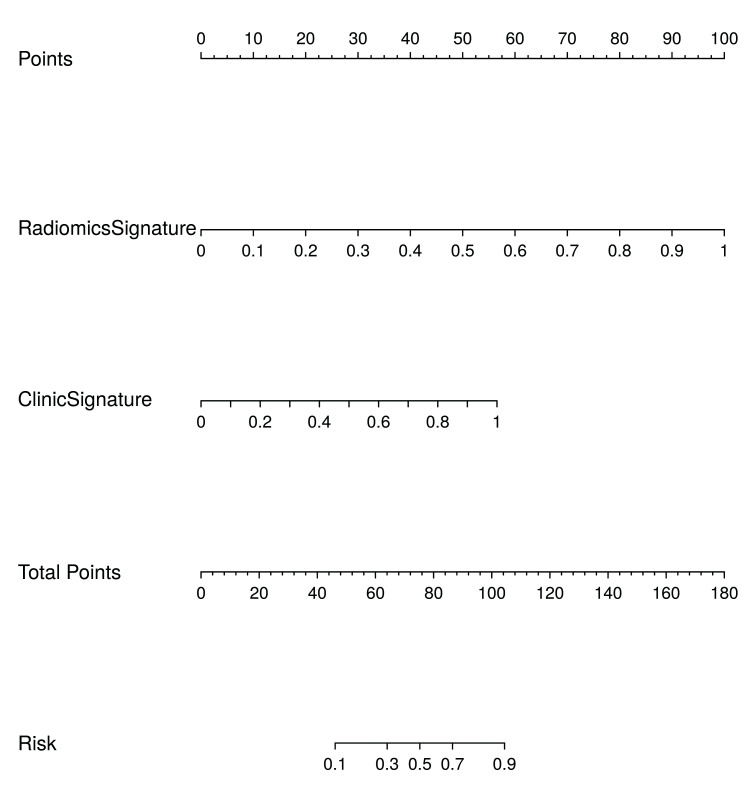
The nomogram model for predicting TBAD, where each level of the predictor variable corresponds to a specific score. The total score is generated by summarizing the scores of each predictor variable. The total score corresponds to the probability size of TBAD.

All radiomics-based models constructed using eight different machine learning algorithms demonstrated strong predictive performance in both the training and validation cohorts, highlighting their potential utility in the early diagnosis of TBAD (Table-II). To visually illustrate the model’s advantages, Python was used to plot the ROC curve for the validation cohort. As shown in Fig. 2, the AUC values of the eight machine-learning-established radiomics models in the validation cohort were all greater than 0.9, indicating that the constructed models exhibited excellent diagnostic accuracy. To better apply radiomics models to clinical work, effectively improve the prediction accuracy of the models, and more intuitively demonstrate the advantages of the models, the clinical model was integrated with the imaging model. A logistic regression was then used to establish and ultimately draw the nomogram of the clinical radiomics model (Fig.3).

**Table-II:** Comparison of Models Constructed by Machine Learning in Train ing cohort and Validation cohort.

No.	model_name	Accuracy	AUC	95% CI	Sensitivity	Specificity	PPV	NPV	Precision	Recall	F1	Threshold
***Training cohort***												
1	LR	0.943	0.987	0.975 - 1.000	0.915	0.971	0.97	0.918	0.97	0.915	0.942	0.616
2	SVM	0.950	0.986	0.970 - 1.0000	0.958	0.942	0.944	0.956	0.944	0.958	0.951	0.518
3	KNN	0.936	0.981	0.965 - 1.000	0.887	0.986	0.984	0.895	0.984	0.887	0.933	0.600
4	RandomForest	1.000	1.000	1.000 - 1.000	1.000	1.000	1.000	1.000	1.000	1.000	1.000	0.500
5	ExtraTrees	1.000	1.000	1.000 - 1.000	1.000	1.000	1.000	1.000	1.000	1.000	1.000	1.000
6	XGBoost	1.000	1.000	1.000 - 1.000	1.000	1.000	1.000	1.000	1.000	1.000	1.000	0.646
7	LightGBM	0.957	0.986	0.972 - 0.999	0.986	0.928	0.933	0.985	0.933	0.986	0.959	0.505
8	MLP	0.936	0.979	0.960 - 0.997	0.887	0.986	0.984	0.895	0.984	0.887	0.933	0.688
***Validation cohort***												
1	LR	0.967	0.973	0.928 - 1.000	1.000	0.935	0.935	1.000	0.935	1.000	0.967	0.510
2	SVM	0.967	0.986	0.962 - 1.000	1.000	0.935	0.935	1.000	0.935	1.000	0.967	0.578
3	KNN	0.933	0.972	0.934 - 1.000	0.966	0.933	0.903	0.966	0.903	0.966	0.933	0.600
4	RandomForest	0.917	0.948	0.886 - 1.000	1.000	0.897	0.853	1.000	0.853	1.000	0.921	0.400
5	ExtraTrees	0.950	0.981	0.956 - 1.000	0.966	0.935	0.933	0.967	0.933	0.966	0.949	0.600
6	XGBoost	0.950	0.972	0.930 - 1.000	0.966	0.935	0.933	0.967	0.933	0.966	0.949	0.735
7	LightGBM	0.917	0.957	0.910 - 1.000	1.000	0.839	0.853	1.000	0.853	1.000	0.921	0.474
8	MLP	0.967	0.973	0.9289 - 1.000	1.000	0.935	0.935	1.000	0.935	1.000	0.967	0.552

**Fig.4:**
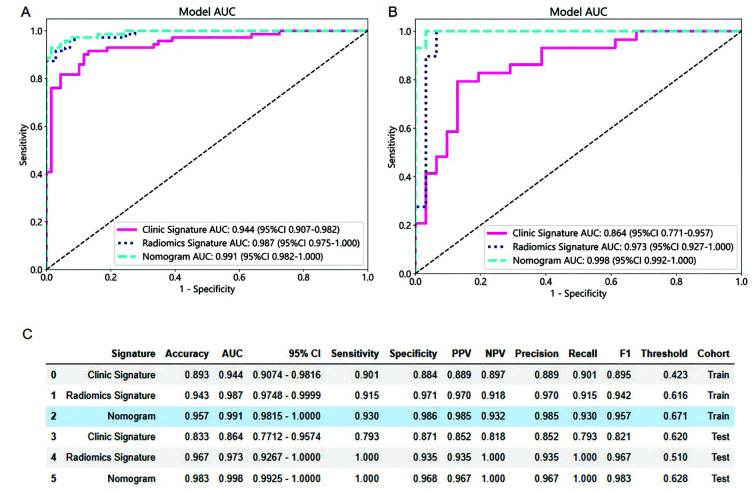
ROC curve. (A) The clinical model, radiomics model, and ROC curve of Nomogram of the training cohort; (B) Verify the clinical model, radiomics model, and ROC curve of Nomogram for the validation set; (C) AUC values of clinical models, radiomics models, and Nomogram.

**Fig.5:**
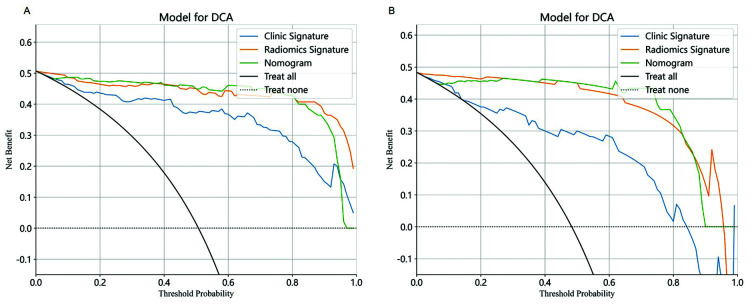
Decision Curve Analysis (DCA) curve. (A) Validation cohort DCA; (B) training cohort DCA. The x-axis of the DCA curve graph represents the values of different probabilities pt, while the y-axis represents the calculated net benefit. The three solid lines represent three different models. The gray solid diagonal line represents the diagonal line with a negative slope, indicating clinical net benefit when TBAD cannot be predicted in extreme cases. The horizontal line at 0 on the vertical axis represents that in extreme cases, 100% prediction of TBAD results in a clinical net benefit rate of 0. It can be seen that the nomogram demonstrated the highest clinical benefit in both the training and validation cohorts.

To evaluate the clinical utility of the nomogram, its performance was compared to that of traditional clinical models and standalone radiomics models derived through machine learning techniques. In the training cohort, the AUC of the nomogram was 0.991, the AUC of the radiomics model was 0.987, and the AUC of the clinical model was 0.944. The results indicated that the nomogram showed better prediction accuracy. In the validation cohort, the AUC of the nomogram was 0.998, the AUC of the radiomics model was 0.973, and the AUC of the clinical model was 0.864, suggesting that the nomogram has a better prediction accuracy (Fig.4). Python software was used to draw the Decision Curve Analysis (DCA) curve. The DCA curve of the nomogram demonstrated its clinical usefulness, which was superior to that of other models (Fig.5).

**Fig.6:**
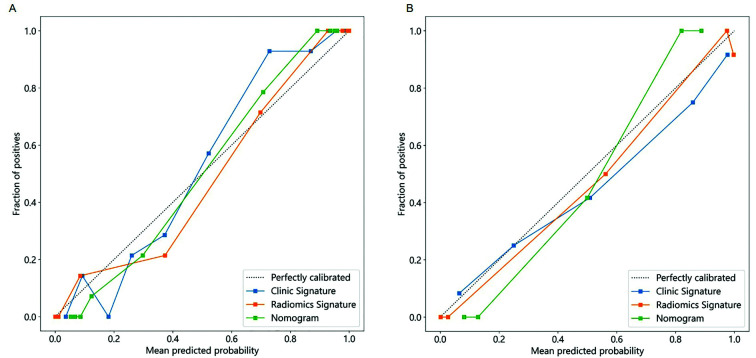
Calibration curve. (A) Calibration chart of the training queue. (B) Verify the calibration hart in the queue. The x-axis represents the predicted probability of TBAD. The y-axis represents the observed TBAD. The diagonal dashed line represents the perfect prediction of the ideal model. The solid line represents the performance of the column chart. It indicates that solid lines are closer to diagonal dashed lines for better prediction.

To fully demonstrate the advantages of the nomogram, calibration curves for three models were then drawn. As shown in Fig.6, the 45 ° dashed line represents the ideal predictive model, while the other three solid lines represent the predictive performance of each model. The closer the solid line is to the dashed line, the higher the model’s accuracy. In both the training cohort and validation cohort, the calibration curves of the nomogram were more closely aligned with the dashed line, indicating that the nomogram exhibited higher predictive accuracy in the early diagnosis of TBAD (Fig.6).

The DeLong test was used to compare the performance of each ROC curve and to assess the differences between different models. In the training cohort, the nomogram demonstrated the best predictive performance, with statistically significant differences compared to the other two models (P < 0.05). In the validation cohort, the nomogram achieved the highest average AUC among all models. Although its performance was superior to that of the clinical model (P < 0.05), the difference between the nomogram and the radiomics-only model was not statistically significant (P = 0.256), as confirmed by the DeLong test (Table III).

**Table-III:** DeLong test.

Cohort	Nomogram vs. Clinical Model	Nomogram vs. Radiomics model	Radiomics model vs. Clinical Model
Training cohort	0.008	0.008	0.026
Validation cohort	0.005	0.256	0.05

## DISCUSSION

This study demonstrated that the predictive model, established based on radiomics analysis of CT images, demonstrates good predictive ability in recognizing TBAD. The basic concept of radiomics, first proposed in 2012, involves extracting a large number of imaging features from radiographic images with high throughput and utilizing automatic or semi-automatic analysis methods to transform imaging data into high-resolution and exploitable spatial data.^11^,^12^ Due to its high efficiency and accuracy, radiomics has gradually been applied in medical research.^11^-^13^ It demonstrated significant clinical value in oncology, from diagnosis and molecular typing to treatment selection, evaluation of prognosis, and prediction of treatment efficacy.^12^,^13^ Multiple teams have since then made significant progress in using radiomics to assist in the diagnosis of rectal cancer and predict the prognosis of lung cancer.^14^,^15^

Kolossvary et al.^16^ first reported differences in radiomics features between the napkin ring and the non-napkin ring signs of coronary arteries. Subsequently, radiomics has been gradually applied to vascular diseases. Zhou et al.^17^ established an impact omics model based on CT plain scan images and demonstrated that this model is faster, has better performance, and higher accuracy compared to experienced radiologists.^17^ Ding et al.^18^ conducted in-depth research on predicting disease progression in intramural hematoma (IMH) patients based on CTA radiomics using machine learning, combining clinical factors and radiological features to construct nomograms. They demonstrated that the final model had good predictive ability for IMH prognosis. Guo et al.^19^ developed a radiomics model based on CT plain scan, which showed excellent predictive performance in screening for thoracic aortic dissection and effectively compensated for the diagnostic errors of radiologists.

This study demonstrated that integrating clinical variables with radiomics features improved model performance, as evidenced by the superior AUC of the nomogram compared to models based on either component alone. This enhancement stems from the complementary nature of the two feature sets: radiomics captures subtle imaging patterns that are beyond human perception, while clinical indicators, such as D-dimer, offer biological context related to disease mechanisms. By employing logistic regression, the model learned optimal weights for each variable, yielding a predictive tool that is both interpretable and statistically robust.

In this study, the radiomics features were extracted from CT non-contrast images, and the LASSO was used to construct dimensionality reduction and prediction models. Out of three models built in this study, the nomogram showed better predictive performance in both the training cohort and validation cohort due to its larger AUC area. To more intuitively demonstrate the advantages brought by the nomogram, the calibration curves were drawn. In both the training and the validation cohorts, the calibration curves of the nomogram were more closely aligned with the dashed line, indicating that the nomogram had higher predictive accuracy in early diagnosis of TBAD. Finally, Python software was used to draw DCA. The validation of the DCA curve confirmed that the nomogram was associated with the highest clinical benefit in both training and validation cohorts.

The calibration curves further confirmed the superior performance of the nomogram. In both the training and validation cohorts, the calibration curves were closely aligned with the ideal reference line, indicating excellent agreement between predicted probabilities and actual outcomes. Additionally, decision curve analysis (DCA) demonstrated that the nomogram provided the highest net clinical benefit across a range of threshold probabilities, reinforcing its potential utility in real-world clinical decision-making. These findings highlight the nomogram’s promise as a reliable tool for early TBAD diagnosis.

The results of this study indicate that the concentration of D-dimer can aid in the diagnosis of TBAD. However, while this index has the advantages of being inexpensive and a fast way of diagnosing TBAD, radiomics demonstrated its diagnostic superiority. In agreement with these results, the studies of Asha et al. and Watanabe et al. also suggest that while D-dimer has significance in the diagnosis of TBAD, it still has disadvantages compared to radiomics models based on CT plain scan.^20^,^21^

One of the key strengths of this study is the use of non-contrast CT imaging, which is widely available and commonly employed in emergency departments. This enhances the clinical accessibility and generalizability of the proposed nomogram. By integrating radiomics features with clinical variables, the model achieved superior diagnostic performance while maintaining interpretability. The reproducible pipeline encompasses automated feature extraction, LASSO-based feature selection, and cross-validated model construction, providing a methodological framework that can be adapted for other vascular pathologies. From a clinical perspective, the nomogram may function as a rapid decision-support tool in emergency and outpatient settings, particularly for patients with atypical symptoms where traditional diagnosis may be delayed. The risk-stratified scores generated by the nomogram can guide downstream diagnostic decisions, such as prioritizing CTA imaging or referring to vascular specialists, thereby facilitating earlier identification and management of TBAD cases.

This study combines clinical and imaging data to construct a nomogram model based on CT plain scan for the diagnosis and prognosis prediction of Type-B aortic dissection. The study employs an interdisciplinary approach that combines medicine and engineering, utilizing rich clinical imaging and data, as well as computer image segmentation, extraction, and modeling methods, to efficiently integrate imaging, clinical medicine, and computer science. This approach offers new tools and ideas for researching and solving complex problems in the medical field.^22^-^24^ Although the nomogram demonstrated high diagnostic performance in internal validation, several limitations may affect its generalizability. First, the model was developed using retrospective data from a single center with a relatively small and imbalanced sample, which may introduce selection bias and limit representativeness. Second, the high feature-to-sample ratio increases the risk of overfitting, despite the use of LASSO regression, stratified sampling, and strict cross-validation procedures to mitigate this. Third, the lack of external validation limits the ability to assess performance across diverse populations and imaging protocols. These limitations underscore the need for future multicenter, prospective studies involving larger and more diverse cohorts. Such studies should incorporate external datasets from different institutions and time periods to rigorously evaluate the robustness and clinical applicability of the nomogram. Additionally, minor performance advantages observed in the nomogram may have been underestimated in prior research due to the limited sample sizes. Future work should aim to expand the clinical dataset, improve radiomics feature engineering, and explore diverse machine learning frameworks to fully realize the predictive potential of the model^23^-^25^ Additionally, the selection of machine learning algorithms -Logistic Regression (LR), Support Vector Machine (SVM), Random Forest (RF), and XGBoost-was guided by their established performance in radiomics tasks with limited sample sizes and their relatively high interpretability in clinical contexts. Ensemble models such as RF and XGBoost achieved perfect AUCs (1.000) in the training cohort. While encouraging, this may indicate overfitting due to their inherent tendency to memorize training data. Therefore, caution is warranted when interpreting these results. To reduce this risk, a stratified random sampling approach was adopted, and feature selection was conducted strictly within the training folds to avoid information leakage. Additionally, performance was validated through 10-fold cross-validation and comparison across multiple algorithms to ensure robustness. However, deep learning approaches, such as convolutional neural networks (CNNs), were not employed due to the relatively small dataset and lack of pixel-level annotations, which are typically required for effective end-to-end training. Furthermore, CNN-based models demand extensive external validation before clinical translation, which was beyond the scope of this single-center, retrospective study. Future studies with larger annotated datasets should explore the potential of deep learning to further enhance diagnostic performance.

### Limitations:.

First, it was based on a relatively small and imbalanced dataset from a single center, which may restrict the generalizability of the findings. Second, the high feature-to-sample ratio (1,561 radiomic features vs. 140 training samples) poses a risk of overfitting, despite the use of LASSO regression and nested cross-validation. Third, no external validation was performed; therefore, the model’s performance remains limited to internal testing. Future studies should incorporate prospective, multicenter cohorts with independent external datasets to enhance robustness and clinical applicability. Additionally, differences in baseline characteristics between TBAD and non-TBAD groups-such as age, D-dimer levels, and comorbidities-may have introduced bias. Although consistent imaging protocols were used and non-TBAD cases without vascular disease were selected, future research should consider stricter matching methods, such as propensity score matching (PSM), to mitigate potential confounding. Lastly, although stratified random sampling was employed to ensure comparability between the training and validation sets, the single-center design may still result in clinical homogeneity. Post hoc analysis confirmed that no significant differences existed in key clinical variables between subsets, supporting the reliability of internal validation results.

## CONCLUSION

This study constructed multiple models to quantitatively diagnose early TBAD. The proposed nomogram, which integrates clinical characteristics, CTA imaging features, and radiomics signatures, demonstrated high diagnostic accuracy in internal validation. This comprehensive model shows considerable potential as a decision-support tool for the early identification of TBAD and may aid clinicians in making timely diagnoses and management decisions. However, the clinical utility of the model needs to be confirmed through large-scale, multicenter external validation before it can be implemented in routine practice.

### Authors’ contributions:

**YS:** Literature search, study design and manuscript writing.

**XS, JN, QJ, GW and JZ:** Data collection, data analysis and interpretation. Critical review.

**YS:** Manuscript revision and validation and is responsible for the integrity of the study. All authors have read and approved the final manuscript.
